# Supplementary material for: TRIM32 and Malin in Neurological and Neuromuscular Rare Diseases
Source: Cells. 2021 Apr 6;10(4):820. doi: 10.3390/cells10040820 (PMC8067510; doi:10.3390/cells10040820)
Supplement: Supplementary file 1 [file cells-10-00820-s001.pdf]

Supplementary Table 1. Reported TRIM32 substrates/interactors listed alphabetically.

Protein	Substrate/Interactor	Outcome	Effect of LGMDR8 mutations	Reference (PMID)
14-3-3	Interactor (NHL)	Prevents TRIM32 auto-ubiquitination and CBs formation. Stabilizes soluble TRIM32.		23444366
α-Actin	Substrate (*in vitro*)	Poly-ubiquitination	N/A	22908310
	Substrate (*in vitro*)	1/2 Ub attached. Reduced levels in HEK with OE TRIM32.	Same as WT (also for auto-ubiquitination)	16243356
α-Actinin	Substrate (*in vitro* soluble muscular fraction. Actual data not shown)	Not shown	N/A	22908310
Abi2	Substrate (NHL)	Poly-ubiquitination and degradation	N/A	18632609
Ago-1	Interactor (NHL)	Enhancement of miRNAs activity	N/A	19269368
	Interactor		N/A	25722370
Ago-2	Interactor		N/A	25722370
	Interactor		N/A	28508149
Ago-3	Interactor		N/A	25722370
Aldolase	Interactor (NHL)	Stabilization (?)	N/A	32223900
AMBRA1	Interactor (RING-BBox)		Not affected	31123703
AMPKa2	Substrate	Poly-Ubiquitination	N/A	21798009
AMPKb2	Substrate	Poly-Ubiquitination	N/A	21798009
ARID1A	Substrate	Ubiquitination and degradation	N/A	31914402
AXIN1	Substrate	Ubiquitination and degradation	N/A	31967859
c-myc	Substrate	Ubiquitination and degradation		19269368
	Substrate	Ubiquitination and degradation	N/A	22299041
DDX6	Interactor	Enhancement of miRNAs activity	N/A	25722370
Desmin	Substrate (*in vitro*)	Poly-ubiquitination and degradation	N/A	22908310; 28096335
Dysbindin	Substrate	Poly-ubiquitination and degradation	N/A	28465353
	Substrate	Poly-Ubiquitination and degradation	D487N and R394H interact but do not ubiquitinate. P130S behaves like WT	19349376
ERK	Interactor		N/A	31621984
Gli1	Substrate (NHL)	Ubiquitination and degradation	N/A	31527798
HSP70	Interactor	Formation of TRIM32 CBs	N/A	28052117
LRRK2	Interactor		N/A	28508149
MycN	Substrate	Ubiquitination and degradation	N/A	25100564
Myosin Heavy Chain	Interactor (Coiled-Coil; *in vitro*)	Not ubiquitinated (*in vitro*)	Same as WT.	16243356
Myosin regulatory light chain	Interactor (*in vitro* soluble muscular fraction)	1/2 Ub attached	N/A	22908310
NDRG2	Substrate	Mainly mono-Ub, faint K48 poly-Ub. Degradation (?)	N/A	25701873
NPHP5	Substrate	K63-linked poly-ubiquitination and delocalization	N/A	28498859
NPHP7/Glis2	Interactor (RING-BBox)	Mixed K48/K63 ubiquitination. Glis2 stabilization.	D487N slightly less active on Glis2. Abolished auto-ubiquitination.	24500717
Oct-4	Substrate	Ubiquitination and degradation	N/A	26307407
Otulin	Substrate (NHL)	K63-linked poly-ubiquitination	R394H, D487N and D588del almost abolish ubiquitination (also self mono-ub)	31504727
p53	Substrate (NHL)	Poly-ubiquitination and degradation	N/A	25146927
p62	Substrate	Mono-ubiquitination □ Enhancement of formation and turnover of p62 puncta	D487N and V591M abolish ubiquitination and oligomerization of p62	31685529
p73	Substrate	Ubiquitination and degradation	N/A	23828567
PB1	Substrate (CC)	K48-linked ubiquitination and degradation	N/A	26057645
Phosphoglycerate mutase 78	Interactor (NHL)	Stabilization (?)	N/A	32223900

Protein	Substrate/Interactor	Outcome	Effect of LGMDR8 mutations	Reference (PMID)
Piasy	Substrate (NHL)	Ubiquitination and degradation	Deficient binding and ubiquitination (D489N)	16816390
PKCζ	Substrate (NHL)	Poly-ubiquitination (and degradation?)	N/A	21732497; 33096083
Pumilio 1	Interactor		N/A	25722370
R5/PTG	Substrate	Poly-Ubiquitination	N/A	21798009
RARα	Substrate	Poly-ubiquitination (stabilization)	N/A	21984809
RGS10	Substrate	Ubiquitination and proteasomal degradation	N/A	31828304
SseK3	Interactor		N/A	26394407
STING	Substrate (NHL)	K63-linked ubiquitination	N/A	22745133
	Substrate	K63-linked ubiquitination	N/A	28954259
Tat (HIV)	Interactor	N/A	N/A	7778269
TRAF2	Substrate	K63-linked ubiquitination	N/A	32145086
TRIF	Interactor	Ubiquitination-independent degradation (lysosomal)	N/A	28898289
TRIM32	Substrate	Mono-ubiquitination	D487N and V591M abolish self-mono-ubiquitination	31685529
	Substrate	Mono/poly-ubiquitination	N/A	14578165
Tropomyosin 1 alpha chain	Substrate (*in vitro* soluble muscular fraction)	1/2 Ub attached	N/A	22908310
UbE2D1, D2, D3, E1, E2, E3, N, V1, V2	Interactor			21143188
ULK1	Interactor (RING-BBox + NHL)	Autophagy activation through unanchored K63 chains	Abolished binding	31123703
USP7	Interactor (RING)		N/A	29899379
XIAP	Substrate (NHL)	Poly-ubiquitination and degradation	N/A	21628460

Supplementary Table 2. Reported malin substrates/interactors listed alphabetically.

Protein	Substrate/interactor	Outcome	Reference (PMID)
AGL	substrate	regulation by ubiquitination	17908927
alpha ARRESTIN1, beta ARRESTIN 1 and 2	interactors	stability of GLT-1	33368637
AMPK subunits alpha and beta	substrate	K63 linked polyubiquitination	20534808
BECLIN, VPS34, VPS15, ATG14L, UVRAG	substrates	impairment of the maturation of autophagosomes	31758957
CHIP	interactor	regulation of the activity of the transcription factor HSF1	21652633
DISHEVELLED2	substrate	K48 and K63 ubiquitination and degradation	22223637
EAAT2/GLT-1	substrate	localization of GLT-1 at the plasma membrane	33368637
GL	substrate	ubiquitination and inhibition of glycogen accumulation	18070875
GLYCOGEN SYNTHASE	substrate	ubiquitination and proteasome-dependent degradation	17952067
LAFORIN	substrate	polyubiquitination and degradation	15930137
NNAT (neuronatin)	interactor	Ubiquitination, proteasomal degradation and regulation of glycogen synthesis	21742036
p62	substrate	Autophagy receptor that targets substrates for autophagy degradation	26546463
PRDM8	interactor	nuclear interaction	22961547
PYRUVATE KINASE M1,M2	substrate	ubiquitination and nuclear translocation only of PKM2	26493215
R5/PTG	substrate	ubiquitination and inhibition of glycogen accumulation	18029386; 17952067; 18070875
R6	substrate	ubiquitination and inhibition of glycogen accumulation	18070875; 23624058
UBE2D1	interactor	E2 enzyme of ubiquitination process	15930137
UBE2D3	interactor	E2 enzyme of ubiquitination process	15930137; 22223637
UBE2E1	interactor	E2 enzyme of ubiquitination process	15930137
UBE2H	interactor	E2 enzyme of ubiquitination process	15930137
UBE2N	interactor	E2 enzyme of ubiquitination process	26546463
